# A How-to-Model Guide for Neuroscience

**DOI:** 10.1523/ENEURO.0352-19.2019

**Published:** 2020-02-14

**Authors:** Gunnar Blohm, Konrad P. Kording, Paul R. Schrater

**Affiliations:** 1 Centre for Neuroscience Research, Queen’s University, Kingston, Ontario K7L 3N6, Canada; 2 Departments of Bioengineering and Neuroscience, University of Pennsylvania, Philadelphia, Pennsylvania 19104; 3 Departments of Psychology and Computer Science, University of Minnesota, Minneapolis, Minnesota 55455

## Abstract

Within neuroscience, models have many roles, including driving hypotheses, making assumptions explicit, synthesizing knowledge, making experimental predictions, and facilitating applications to medicine. While specific modeling techniques are often taught, the process of constructing models for a given phenomenon or question is generally left opaque. Here, informed by guiding many students through modeling exercises at our summer school in CoSMo (Computational Sensory-Motor Neuroscience), we provide a practical 10-step breakdown of the modeling process. This approach makes choices and criteria more explicit and replicable. Experiment design has long been taught in neuroscience; the modeling process should receive the same attention.

## Significance Statement

Modeling in neuroscience is often perceived as a mysterious process and is hard to teach. Here we provide the first how-to-model guide that breaks down the modeling endeavor into a step-by-step process.

## Introduction

The development of models is an integral part of neuroscience and related disciplines, such as psychology, kinesiology, and cognitive science. Models can provide unique and useful insights. For example, computational models are used to compactly describe large amounts of data. Models are often used to obtain causal claims about the relation between neural properties and behavior. They make predictions and can thus allow more targeted experiments. Models allow virtual experimentation, making it easier to get intuitions. Models also force scientists to make their assumptions explicit, which makes scientific communication more precise. Finally, models can lead to applications across science, health care, and technology (e.g., one can plan interventions by simulating their impact on brain and behavior). Model-driven approaches thus accelerate progress across clinical and basic research.

There are countless models in neuroscience, and for each modeling technique we can find an article describing how it is constructed. For the more popular techniques, we can find textbooks that describe the mechanics of constructing and testing models, pitfalls, tips and tricks usually tailored to the particular types of data, and questions that made the technique popular. However, when approaching new questions, new data types, or different scientific goals and objectives, it is unclear how to start. Confronted with a phenomenon and a scientific goal, every researcher is faced with a difficult set of questions. Which concepts should we use? Which mathematical framework (i.e., technique)? Which code? What should the overall logic be? All these questions are currently unarticulated and hidden in the scientific training process, and students implicitly learn approaches across neuroscience through imitation and mentoring. While this can be an effective way of transmitting modeling techniques for ongoing questions, it is an ineffective way to train students to innovate, competently address new problems, or synthesize and extend methods. Instead, there should be a clearly structured thought process that clearly identifies how the phenomenon along with the goals of modeling give rise to the ultimate models. What is missing is a procedure by which we can address a phenomenon with modeling in a way that brings us closer to our scientific goals.

We have observed many students learning how to build models during our 8 year experience with CoSMo (summer school in Computational Sensory-Motor Neuroscience; www.compneurosci.com/CoSMo) where we taught students from senior undergraduates to seasoned researchers how to model. Through teaching and project work, we have tried to convey to them the process of constructing models from scratch. All three of the authors are also building models, and importantly we cover a broad range of types of modeling. This includes machine learning, Bayesian modeling, linear systems modeling, realistic muscle modeling, spiking neural networks, and single-cell models. In addition, we have brought dozens of leading computational neuroscientists as guest lecturers in the course, providing us with template examples of a broad array of successful modeling approaches applied to a diverse set of phenomena and questions. As such, we feel that we have experienced the model construction process in a uniquely cross-cutting way. While the modeling process is complex and multifaceted, we believe it can be formalized and made explicit.

Here, we propose a pipeline to modeling that breaks the whole enterprise down into a series of (sometimes interdependent) decision processes. Note that the approach outlined here is not the only way to approach the modeling exercise; rather, it represents one possible systematic, step-by-step approach that—if conducted carefully—should leave little room for failure. By using this approach, we have directed hundreds of CoSMo students in small groups through the full “from scratch” modeling process to successful conclusions in just 2 weeks.

## 10 steps to modeling

**Figure 1.**
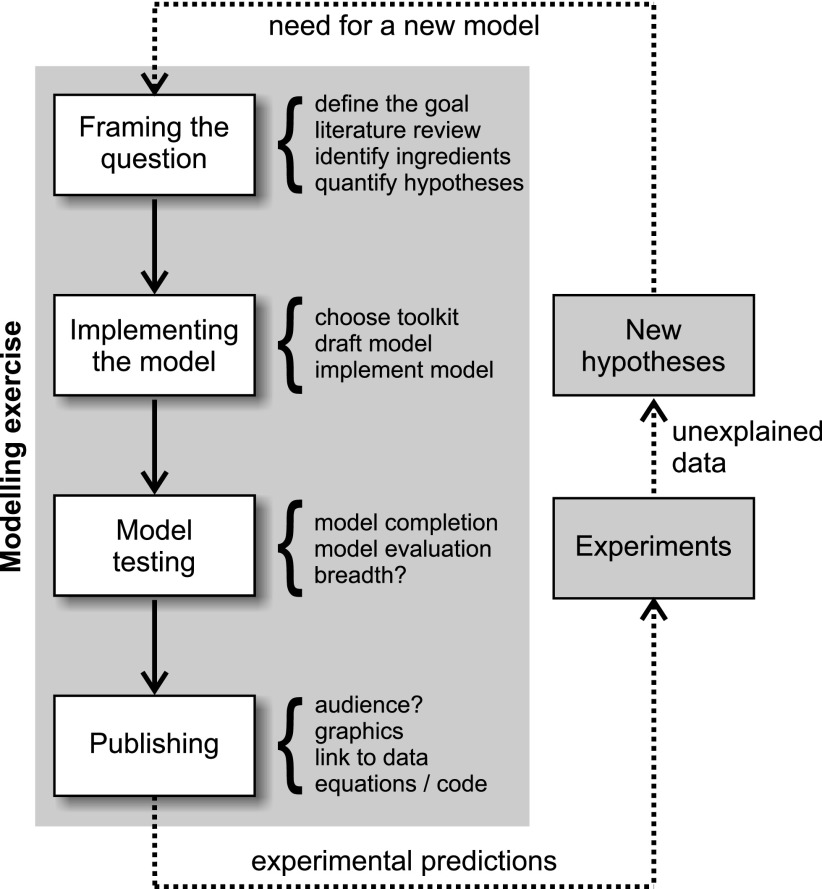
The modeling exercise. Models interact with experiments through the generation of novel model-based experimental predictions. Experimental data will in turn provide new unexplained data and hypotheses that call for new or refined models. Note that modelers do not necessarily need to test their own experimental predictions or collect their own unexplained data; but good modelers should interact with experimentalists. Many good experiments come from modelers annoyingly asking for data.

**Figure 2.**
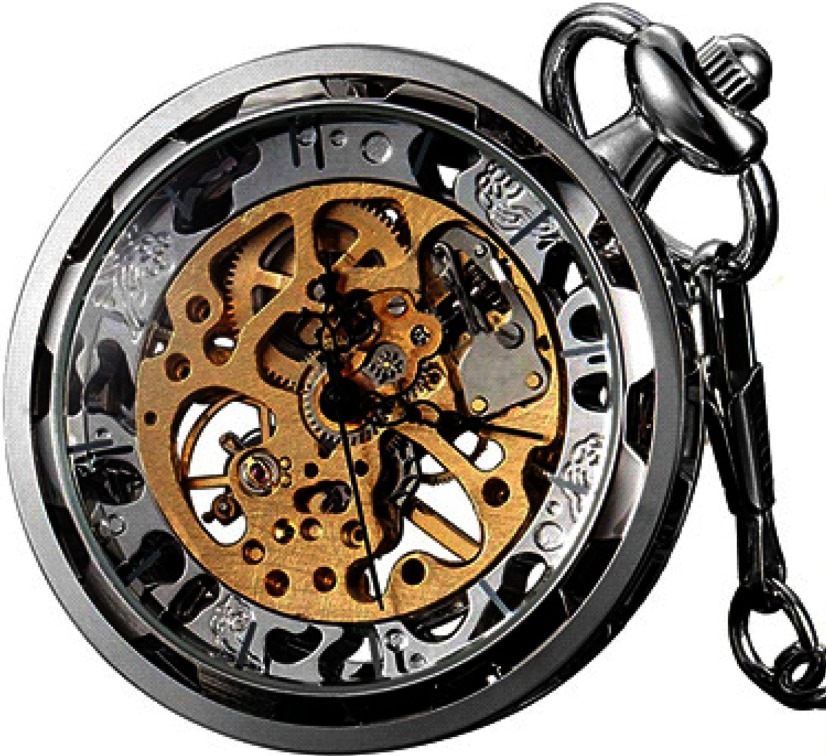
Mechanical watch. Even knowing what it does, its inner workings are far from trivial. Imagine an archeologist finding one of those and not knowing what this is for.

We will suppose that the modeler knows the phenomenon of interest, and has data or specific observations that need to be explained. A good modeling approach needs a good phenomenon to describe. Below, we will highlight a modeling process that consists of 10 main steps, grouped into the following four sections (Fig. 1): framing the question; implementing the model; model testing; and publishing the model. Throughout the discussion, we will use a common example phenomenon that will be well known to most of our readers: Assume that we did not have clocks and ignored that they once existed. Now imagine what an archeologist finding a clock (such as the one in Fig. 2) would go through to find out what it was for. Similar to the Antikythera mechanism (https://en.wikipedia.org/wiki/Antikythera_mechanism), we would have to build a model to explain certain aspects of the observed clock behavior. As such, the clock device in analogy to the brain computes something and we are trying to figure out what it is. We will discuss how we might model the movement of “hands” across the markings of an analog clock. We will go through all the modeling steps as part of a modeling process with which we could address the movement of the digits of the clock. With this example, we will be able to highlight all 10 steps of the modeling process.

### Framing the question

#### Step 1: finding a phenomenon and a question to ask about it

The starting point to all models is a question related to a phenomenon of interest. Thus, the first practical step for the modeler is to build a list or table of the critical observations (Table 1) that define the interesting aspects of the phenomenon—what sets it apart from other things or for which we lack good explanations. For the movement of the clock, the distinguishing features for us are as follows: they are precise, they are circularly periodic, and multiple hands have nested periodicities. These periodicities are approximate multiples of another precisely timed phenomenon—the rotation of the earth. Defining the precise phenomenon is critical to asking a good question.

**Table 1:** Example of critical, distinguishing observations of the clock

Question-answer	What	How	Why
Phenomenon			
1 Tick/s	Loud for 100 ms then silent for 900 ms	Whatever that thingamajig is called	Timekeeping. Duh.
Gears exist	Notches on circles	More notches = slower rotation	Translate faster rotation / slower rotation
1 Rotation/h	Periodicity	120 notches on hour ring, 2 on second ring	Because an hour is a useful division of the day

Once we have characterized the phenomenon, we need to define a meaningful modeling question. It helps to get clarity on the type of question we are asking: are the characteristics that define the “what” of the phenomenon well formed, or do we need to better describe the data? Do we want to ask “how” something works? Or are we interested in “why” the phenomenon exists in the first place? The observations about the clock can lead to different types of questions. What is the relation between the gears and the movement of the arms? How do the gears produce the observed pattern of arm motions? And why would anyone build such a mechanism (Table 1)? None of these questions is inherently more or less interesting; they could all represent legitimate goals for a model builder (Kording et al., 2018). However, a clear choice of such a goal is essential to allow meaningful models. Clearly specify your goal at the outset, to yourself and in all your communication about the modeling project.

Once we have a general phenomenon and question in mind, we can demarcate which aspects of the data our model should capture. Without an exact question, chances are high that one will get lost in the vast oceans of the unknown. This leads to our first dictum of modeling: beginning with the question, write everything down in a precise way! Imprecise questions lead to rapid failure. “Model a clock” would be a bad definition of a goal; after all, it does not identify key observations or criteria for success or failure. The question “How do the angles of the hands predict the time of the day?,” on the other hand, would be a well defined question. It both specifies the phenomenon (time of day relation) and implies criteria for success (low variance at predicting the time of the day).

At this step, it is also helpful to identify aspects of observations that the model will not address to answer the question [e.g., we may decide that we do not (for the current model) care about the mechanisms in the clock]. By focusing on distinguishing features of the phenomena together with intuitions about the factors that should be included in an explanation, the model is focused both on a concrete question and on an appropriate level of abstraction. By maintaining focus, we avoid the inevitable “mission creep” that results from having a fuzzy question; fuzzy questions inevitably pull researchers toward attempting to answer a much larger family of apparently related questions. Having focus also provides a natural Occam’s razor quality to our models. Through focus, our models address the knowledge gap central to the question while minimizing the complexity of the approach.

As part of the objective, the model evaluation method must also be defined. This leads to our second modeling dictum: “Know when to stop!” A well defined modeling goal must have a well defined stopping criteria, or else we will suffer endless mission creep. We should be able to answer the following questions. When are we satisfied with the new model? What would it mean for a model to be better than another model for our criterion? These are difficult questions, but there are clear desiderata that good evaluation criteria should adhere to. The evaluation must ensure the model incorporates the critical observations. The evaluation must make the model connect with actual or potential data. In the clock example, we might wish to reproduce the observed periodicity with a low error. Or we might want to provide an explanation of why there are so many gears and what they are good for. Thinking about a specific experiment that could potentially answer the questions posed is often tremendously useful to ensure that these desiderata are satisfied. It provides a specific, tangible, and intuitive instantiation of an abstract question. Moreover, it inherently provides a benchmark goal for the model to be designed. Indeed, the model should be able to simulate this exact experiment to provide a model-based answer. In the clock, removing a gear or changing a gear ratio could be a good experiment to test the role of gears. Being able to simulate results from a hypothetical experiment or real experiment thus becomes part of the modeling objective.

Finally, it is also important to determine precise evaluation criteria based on well defined qualitative and/or quantitative properties the model should exhibit. This is crucial because data derived from experimental observations is naturally variable, and thus determining criteria that allow us to judge the performance of the model is important to ultimately determine when the modeling exercise is accomplished. For example, is the goal to reproduce general trends/tendencies, or is a detailed match of model and data of importance? Are there certain specific experimental effects or relationships that the model must reproduce? How will performance be measured? For example, if the clock is really meant for timekeeping, then a model of the clock should match its periodicity very closely (i.e., within measurement noise). We will further elaborate on the model evaluation in the Model testing section (Steps 8 and 9). Establishing the evaluation method right from the start will ensure a fair, critical evaluation of the modeling effort and a timely finalization of the model.

In our experience, Step 1 is the most difficult for both novice and experienced researchers. It is the step that requires the most thought, and it is a step often revisited for refinement after realizing that the subsequent steps are not working.

#### Step 2: understanding the state of the art

Before diving into the modeling itself, it is obviously essential to survey the literature. This survey serves to provide additional information about the phenomenon, if there is controversy or specific conditions under which it occurs, and provides background on the set of questions that have already been addressed. From a modeling perspective, it provides insight into the types of abstractions and approaches that might have already been used. What has already been done in terms of modeling? Are there previous models that one can use as a starting point? What hypotheses have other researchers (theoreticians and experimentalists alike) emitted regarding the phenomenon in question? Are there any alternative and/or complementary models or explanations? In the clock example, we may know the elementary theory from school that if we have a gear with N cogs and another with K cogs, then it translates the rotation speed as N/K. This second step will ensure that no important aspects (theoretical and experimental) related to the model are accidentally omitted. It will also provide the specific datasets and/or alternative models to compare the new model against. In addition, this review might provide insight as to the specific evaluation criteria (e.g., root mean square fit error) that are typically used in the field. A literature survey should thus always be conducted prior to building a new model.

It is also important to gain an intuitive and practical understanding of previously proposed models and theories. Such an understanding can only be obtained by reimplementing previous models and exploring their potential and limitations in a hands-on fashion. Exploring previous models familiarizes the researcher with specific approaches, toolkits, and mechanisms that have previously been proposed. Exploring strengths and weaknesses of existing models will help identify and justify the need for a new model. Step 2 can be characterized as a foraging task where the researcher better characterizes the phenomenon, the explanatory gap, and gathers together a set of possibly useful ingredients into the modeler’s workshop, such as concepts, methods, and mechanism.

Finally, the literature review should also allow determination of the skill set needed in order to understand previous modeling endeavors. This could result in the need to learn new skills, whether or not those skills will also be helpful for building the new model. Thus, a good understanding of the state of the art of a field is instrumental to understanding previous models and proposing a new model in the light of previous work. However, our question-centric approach eschews premature adoption of any of these approaches. Instead, we advocate evaluating previous approaches through the lens of the focused question and its basic ingredients.

#### Step 3: determining the basic ingredients

After defining phenomena and objectives, we can now become a bit more specific. Every modeling effort starts with an intuition that will provide an inventory of specific concepts and/or interactions that need to be instantiated. What variables and/or parameters in the question and inventory are needed in the model? Are those constants or do they change over, for instance, space, time, or conditions? Are there any concepts (e.g., value, utility, uncertainty, cost, salience, goals, strategy, plant, dynamics) that need to be instantiated as variables? Can these variables be observed/measured directly or are they latent (internal) variables in the model? In order to instantiate latent variables, they should be related to potential measurements, whether practically possible or not. In our clock example, the angular speed of the gears (latent variable) might matter in determining the movement of the arms (observed), and we know it is constant for a given gear but different across gears. What details can be omitted (e.g., materials the clock is made of)? What are the constraints, initial conditions? How are these variables expected to interact? For example, there is a specific relationship between gear speeds in the clock that is constant and determined by the fraction of number of cogs. What should be the inputs (potentially under experimental control) and outputs (that could be measured; i.e., outputs should typically be the same as the data the model addresses) of the model? Answering these questions will set up the elements that are required in the model as well as the specific conditions that have to be satisfied by the model.

A second much more difficult to acquire—but crucial—set of instruments for the modeler is a library of potential explanatory mechanisms. Such a library is usually collected over time by hands-on exploration of different models, approaches, pieces of math, and algorithms. This goes hand in hand with building an intuition for a research field through exploratory data/model analysis and careful reading of the relevant literature. An intuition is then formed as a result of experiences with different model classes and data. For the clock example, models that produce oscillatory behavior (i.e., periodicity) might be of particular interest. We claim that there is no way around this learning by doing the step (and regular cataloguing of this explanatory set should be a priority for the community). But as a result, the potential required explanatory mechanisms will also help in providing specific concepts and interactions that need to be instantiated. Once the model ingredients and potential mechanisms have been identified, specific hypotheses can be expressed in mathematical language.

#### Step 4: formulating specific, mathematically defined hypotheses

Contrary to the question asked in Step 1, hypotheses propose a specific relationship that could explain a given phenomenon. To formulate a hypothesis in modeling terms, we need to map our intuitions and proposals about mechanisms and variables into precise mathematical language. In this sense, a model is a mathematical quantification of verbal hypotheses. The first step in achieving this is to relate the ingredients identified in Step 3 by quantifying specific hypotheses. For example, the 60:1 ratio of periodicities between the smaller hands of the clock corresponds to tracking seconds/minute. These hypotheses can be expressed in terms of relations between variables and restate the original question from Step 1 in the form of relations between variables, mediated by hypothesized mechanisms and interactions. Thus, these hypotheses are the ones that are identified from the original question and ask: what is the model mechanism expected to do? How are the different parameters expected to influence the model results? Answering these questions with words/sentences will set the modeler up to start expressing relationships between parameters and variables in mathematical language.

Going back to our clock example and supposing we do not know what this device is for, a series of hypotheses can be emitted related to the what, how, and why questions. First, we can hypothesize that the gears will lead to different arm speeds. Second, it is the exact gear ratio that is of importance, and this gear ratio is determined by the dynamics of the spring–balance wheel system. Third, we can hypothesize that clocks are there as timekeeping machines. For all of these hypotheses, we have made use of our inventory of observations about the movement of the arms, the gears, the spring, and the balance wheel. We also need to keep in mind the use of the clock (i.e., people use the clock for scheduling purposes and to regulate/coordinate human behavior. These verbal hypotheses represent the starting point for mathematical abstraction, identifying key components and concepts needed for each question.

Once the hypotheses are spelled out, variable names should be assigned so that hypotheses can be expressed succinctly in those terms. What mathematical relationships are expected? It is good to be explicit here: for example, *y*(*t*) = *f*(*x*(*t*), *k*), but *z*(*t*) does not influence *y*. Can we hypothesize anything about the form of *f*? One advantage of this explicit mathematical notation is that it is also made clear that *x*, *y*, and *z* change over time, while *k* is a constant. Constraints, initial conditions, and any other known or expected relationships can be expressed in a similar way. In our clock example, we can first write that angular velocity of the slowest hand is *vx* = *f*(*r*,*v*0), where *r* is the gear ratio and *v*0 is the resulting speed of the spring–balance wheel system driving the gears (latent variable); we hypothesize *f* to be linear. We can further write a relationship for the gear ratio *r* and hypothesize that the gear ratio between two arms determines their relative angular speed. Let *z*(*t*) be the angle of the fastest hand, *y*(*t*) the intermediate, and *x*(*t*) the slowest hand. Then we hypothesize *vy* = *r* * *vx*, thus the angular position *y*(*t*) = *r* * *x*(*t*) + constant mod 2 * pi, and as above, *y*(*t*) is not influenced by *z*(*t*), rather the converse is true. The spring–balance wheel system should act like a harmonic oscillator determining *v*0 [i.e., *v*0 = *f*(*m*,*k*) where *m* is the mass of the balance wheel and *k* is the spring constant]. Formulating hypotheses for the why question is also possible. If it is indeed a timekeeping machine used to organize human activities (as opposed to a similar looking astronomical position tracker such as the Antikythera mechanism, for example), then there should be a correlation between different peoples’ behavior that is based on their consultation of the clock (and no correlation if it was an astronomical or other device). In that case, we could write that the clock-based behavioral event times *Ti* between different people should be highly correlated (i.e., *Ti* = *f*(*Tj*) for *j* ≠ *i*, where *f* is expected to be linear and with a slope of 1. The resulting mathematical relationships constitute the first step of abstraction that will determine the model approach and identify the model ingredients needed. In addition, these hypotheses will later be evaluated against model behavior. Last, translating the specific hypotheses into mathematical language will ultimately also help in “selling” the model to the research community. Indeed, the more precise the hypotheses, the better the modeling approach can be justified.

**Figure 3.**
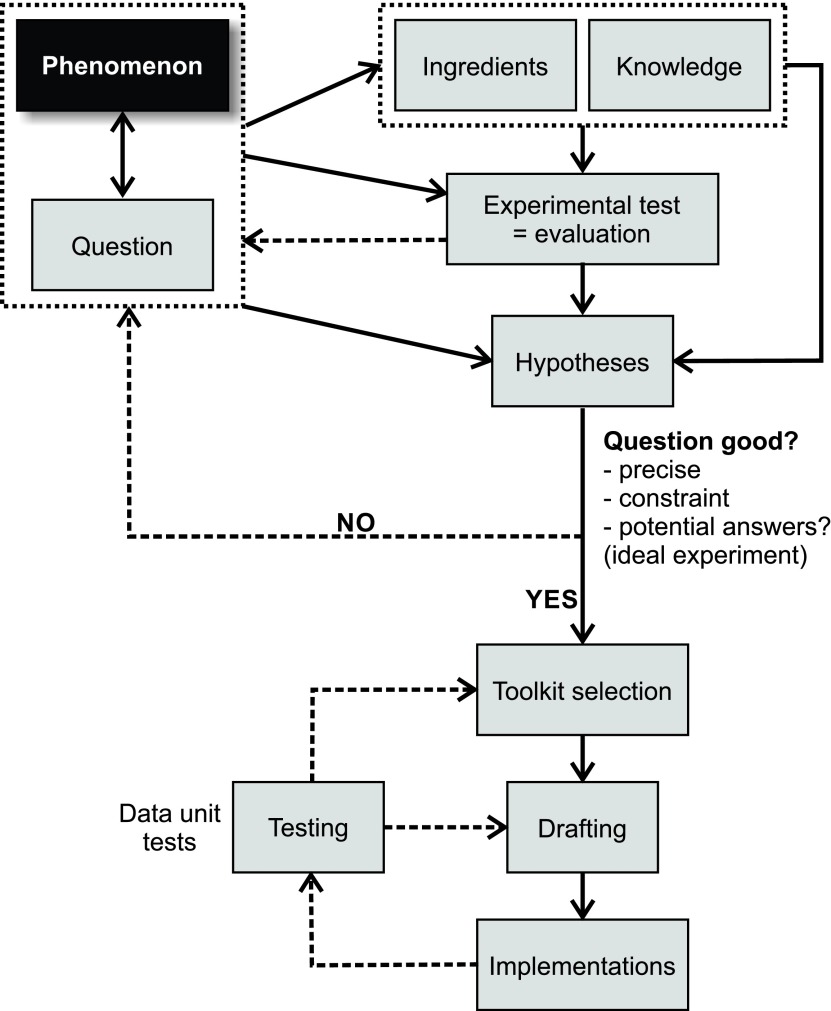
Iterative view of the first steps of the modeling exercise. Consecutive thought processes often identify lack, omissions, imprecisions, and uncertainties that require the modeler to go back and refine their thoughts. This is true when framing the question and independently applies during model implementation. Note that these two processes are serial. One should not start the implementation process without having fully satisfied all model framing criteria and steps. Solid arrows denote direct transitions/dependencies; dashed arrows stand for iterative reconsideration. Once a phenomenon/question is identified, required ingredients and literature review are conducted, which ideally leads to a potential experimental test. If no such test can be found, maybe the question needs reformulating. One should be able to identify specific hypotheses; otherwise there is a lack of specificity/precision in the question that needs to be revisited. Toolkit selection, drafting, and implementation of the model involve iterative unit testing. Unit testing can identify pitfalls in drafting or even in the choice of the toolkit (less frequently) that require adjustment of the model plan.

Finally, it should be noted that Steps 1–4 are linear in an ideal case scenario, but often need to be conducted iteratively (Fig. 3). Indeed, every step has the potential to unmask a weak, imprecise, already answered, not interesting, or too ambitious question. In that case, the original question has to be modified, adapted, clarified, or changed altogether, after which all following steps require reconsideration. This can also happen at later stages during the modeling exercise, but if Steps 1–4 are conducted properly, this should be much less likely to happen. We are now at the point where the practical modeling can begin.

### Implementing the model

#### Step 5: selecting the toolkit

Once the modeling goals are set and the hypotheses are quantified, the most appropriate modeling approach to address the question needs to be selected. It is important to state that different model toolkits can potentially provide an answer to the same question asked. But not all toolkits are equivalent; quite the opposite. Indeed, different toolkits afford answering different types of questions, such as being able to extrapolate versus finding mechanistic reasons for a given phenomenon. Important considerations are: what modeling tools should be used (e.g., mechanics) and what level of abstraction (e.g., what is the purpose of this device) is appropriate? Based on the hypotheses and goals, this should now be relatively easy to do. In the clock example, we might not care about the material properties of the gears but only the number of teeth in the gears. We also cannot lump all gears together because they activate different arms. As a general rule, the model should stay as high-level/abstract as possible, but be as detailed as necessary (Occam’s razor; Feldman, 2016; Seiradakis and Edmunds, 2018). The choice of a modeling toolkit then allows the production of a real model.

Determining which toolkit to use can be far from trivial and requires prior knowledge about the toolkit. As a guideline, a good question to ask is how flexible the toolkit is in terms of behavior. There is no “right” tool, and often there is more than one option to choose from. Tools should interface with data that the model is trying to address. For example, if data consist of changing time series, then the toolkit has to have a dynamic component that can reproduce those time-dependent signal changes. If we are interested in understanding the spring–balance wheel and gear mechanism of the clock, we might turn toward mechanical finite element toolkits to understand how the physical properties of these elements influence the functioning of the clock; or we could just care about the resulting clock arm dynamics and use higher-level kinematics tools instead. Toolkit selection supposes a good knowledge of what the strengths and limitations of each available toolkit are. Preference should be given to toolkits that have more flexibility, span a wider range of behaviors, and are potentially lumpable (i.e., can be reduced in size by using techniques such as population averaging or state-space reductions; e.g., neural networks span a large range of behaviors but lumping is hard). On the other hand, linear systems theory lumps well but does not have the same level of detail as neural networks (but see Eliasmith and Anderson, 2004 for one particular way to do that). In summary: knowledge is key.

Choosing the toolkit also means determining how the model will be solved (i.e., simulated). For example, can an analytical solution be computed or is numerical integration of equations required? If numerical integration is needed, what is, for example, the temporal or spatial resolution? In the eye movement literature, many models make use of the Laplace transform of dynamical systems; this would require learning about the Laplace formalism and how to use it. Here, we will assume that a way to solve the equations of the chosen toolkit can be found. This requires, of course, knowledge about the appropriate fields of physics, mathematics, or computer science, if applicable, and it is very difficult to succeed as a modeler without such an appropriate background.

#### Step 6: planning the model

**Figure 4.**
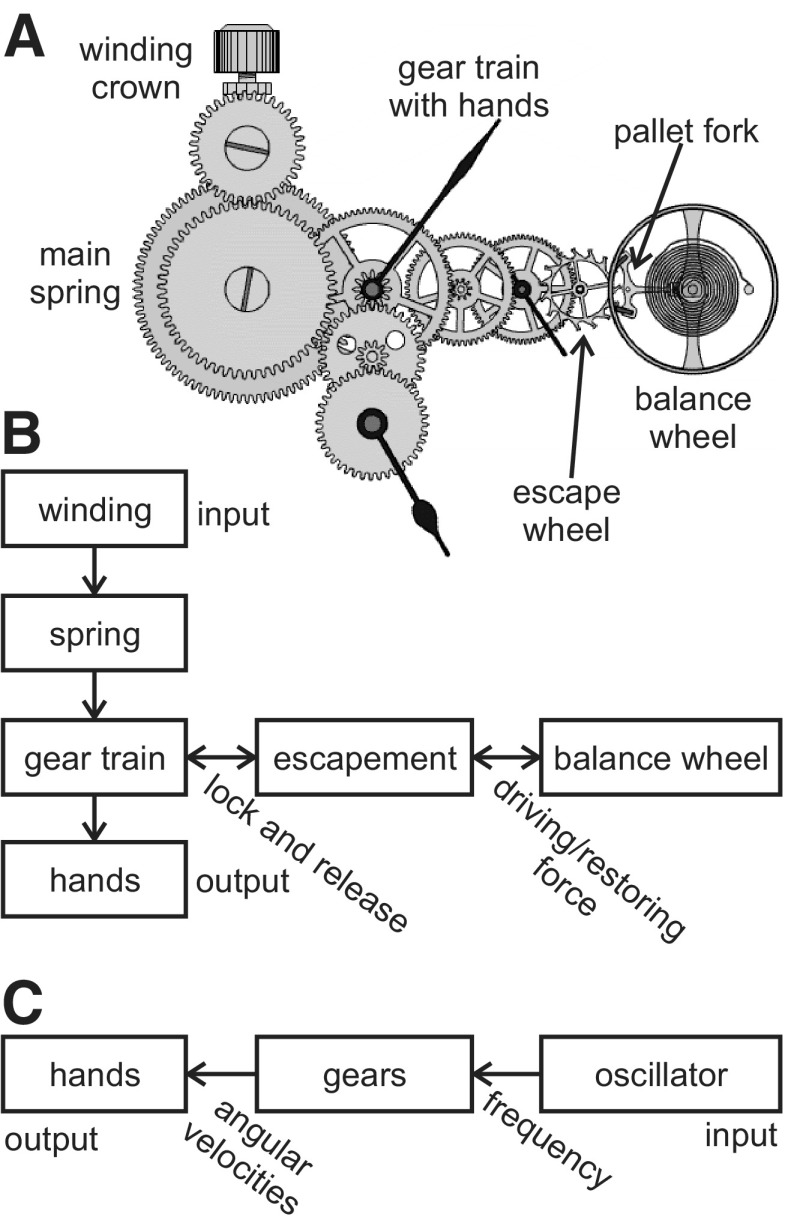
Model diagrams. ***A***, Mechanical elements of a mechanical clock. ***B***, Flow diagram equivalent of the mechanical clock. ***C***, Dynamical systems equivalent to the mechanical clock. ***B***, ***C***, Note: inputs are different between full model (***B***) and reduced model (***C***). Exemplary variables (tilted text) passed between model elements also differ in nature.

We are now ready to start building up the model. This is the point where diagrams are drawn, sketches can be made, equations are formalized and preliminary pieces of code are written. The goal of this step is to put all the components of the hypothesized relationships and explanations in place. As the most important rule, the model should always be kept as simple as possible! It is advised to start with a first draft of the model on paper. All toolkits allow for a graphical representation to be built, but the nature of these drawings can be quite different. For example, a mechanical model of the clock (Fig. 4*A*,*B*) will look different from a dynamical systems description (Fig. 4*C*) of the clock movements, including potentially different inputs (such as in Fig. 4), latent variables, constants, initial conditions, and outputs. Draw out the model components and how they connect to each other/influence one another. This flow diagram (Fig. 4*B*,*C*) will help to organize the equations. It will allow explicit indication of which variables “flow” from one model component to the next. This model diagram will set up the basic components that are expected to be required in the model.

Now each model box, icon, or flow can be considered individually, and its internal workings should be drafted in terms of mathematical equations. These should be explicit equations that can later be implemented in simulation programs. In case of the clock example, the gear train box might be subdivided into one functional box for each gear in the flow diagram determining the equations of motion of the gear and relating the input of the boxes (the angular velocity of the previous gear) to the output (angular velocity of this gear). It can require extensive work to identify the appropriate mathematical relationships, equations, and formalisms. But at this stage, filling the boxes, quantifying the icons, and/or specifying the interactions between them should be relatively easy since the basic input and output variables of the subsystems of the model have already been defined and the modeler’s goal is thus “only” is to relate those variables. It is important to keep in mind here that a model must include a way to relate model variables to measurements. Otherwise, the modeling exercise will typically feel pointless. Ultimately, the drafting process will result in a first model on paper that is ready to be implemented and might become a model diagram in a subsequent publication.

#### Step 7: implementing the model

The model is now ready to be implemented. This means that computer simulations can be set up and run and/or analytical solutions can be found. Each box, icon, or flow relationship identified in Step 6 should be implemented separately and tested or understood individually before connecting it into the overall model. This “unit test” procedure will ensure the functionality of the individual components before evaluating the more complex behavior of the full model.

Individual model components can then be combined. If there are any alternatives or uncertainties, it is advised to start with the easiest implementation of the model or of part of the model and to test its functionality along the way. A general guideline is to build up the model step by step and test its function at each step. Starting with a simple version of the model and progressively adding all the elements will not only produce an understanding of what simpler models can do, but also minimize errors in construction. Moreover, playing with all the components of the model on implementation time can provide deep insights into the way they actually work. In our clock example, there are gears for rewinding the spring mechanisms of the clock. Those gears can be modeled, but they will not influence the movement of the arms (unless the spring is loose, of course). Thus, these rewinding gears are not crucial for the timekeeping function of the clock mechanism and can be left out if that kind of understanding is our goal. Answering the question of why a certain model component is crucially needed will ultimately allow justifying the model architecture during the publication process. This process should be continued until the model has been fully implemented.

Once we have implemented a model, we want to make sure we properly understand our own implementation. This makes it necessary to deeply analyze its behavior (Otto and Day, 2011). We should plot model behavior as a function of model parameters. We can analyze model stability/equilibrium points. We can ask how similar the model performs to known models (e.g., those that can be analytically solved). Each modeling toolkit usually comes hand in hand with a set of model analysis tools; details about the latter can be found in the specific toolkit literature. All of these steps may help us in finding mistakes in our model implementation.

### Model testing

#### Step 8: completing the model

One of the hardest questions in modeling is to decide when to stop improving the model and call it final. Referring back to the goals (Step 1) and hypotheses (Step 4) is crucial here. Does the model answer the original question sufficiently (i.e., with enough detail to advance knowledge in the field of study)? Equally importantly, does the model satisfy the evaluation criteria that have been determined prior to building the model? Does it speak to the hypotheses, either confirming or invalidating them? In other words, can the model produce the parametric relationships that have been hypothesized in Step 4? If the answer to all these questions is “yes,” then the modeling exercise might be done. If the original goal has not been met, then the modeler may need to get back to the drawing board.

We need to be mindful on finishing a project when the time has come. On the one hand, we can usually improve model fits; on the other hand, we do that at the risk of overfitting the data we have. Occam’s razor might help here to determine whether it is worth considering more complicated models with more parameters that are perhaps irrelevant or uninterpretable in order to obtain a better fit to the data. The cost of such more complicated models is always the reduced explanatory power. This is mathematically quantified in measures such as the Akaike information criterion, as explained in the following step.

#### Step 9: testing and evaluating the model

In Steps 1 and 4, we set up goals/hypotheses and objectives for our modeling approach. Once we have implemented and tested the model, we can now evaluate how well we did in the modeling approach. How to evaluate how well a model did supremely depends on the nature of the goals. For example, if we only care about the relation between the second and the minute digit of the clock, then explaining their relative movement well would be sufficient. If we want to answer to why clocks exist, our answer would have to look very different. The objectives we defined further up determine how exactly a model is to be evaluated.

However, many different modeling approaches are aimed at describing data. This generally leads to a statistical problem—how can we ask which model better describes the data. Statistics has given us many tools to ask this question. These range from the mean squared error, to methods that correct for the number of free parameters (e.g., the Akaike information criterion) to the ability to predict new and unseen data. Model comparison is a centerpiece in the modern modeling enterprise. Indeed, model comparison is useful to compare a new model against existing precursors/alternatives. It is also often useful to build a class of models instead of just creating one specific instance, in which case model comparison is often used as a means of selecting the best model among the class of models proposed.

Finally, it is important to ask questions about generalizability. The model explains the phenomenon we set out to describe. But knowing this is not enough. Will the model also adequately describe similar situations? Can what we learned from one clock generalize to others? Without quantifying generalization, it is unclear how valuable a model is, and no modeling study should be finished without asking the generalization question.[Boxed-text box1]

Debunking myths:

- Models are not built to win a beauty contest but to explore the unknown.

- Modeling is not a grade school art show: multiplying evaluation criteria to find one in which your model succeeds is not a good idea.

- The model that best fits your data may not be the best model (e.g., because of overfitting and limits to your data).

- Modeling is not a fashion show: models should not be judged in terms of fashionable concepts and mechanisms.

- Models are not your children. Even if you have created them, diapered them, and trained them, do not be a parent protecting your model at all costs, but accept if they fail. After all, it’s meant to fail! The question is how much can we learn from it and how much can it advance knowledge until it fails.

- Do not be a model bigot. You should not just hate a model because it uses a different language than you would use.

Understand what they say first! Irrational toolkit preference is inappropriate and hinders knowledge advancement. Do not judge the mechanic by their toolkit but by what she/he can do with it!

### Publishing

#### Step 10: publishing models

Once everything has been done right, the model has been built, simulations are running, and satisfactory results have been obtained, the goal is to communicate those findings through a scientific publication. This is a tricky exercise in itself, and it is worth spending a few words highlighting aspects that will much improve the likelihood of acceptance. In addition, this section should be a guideline equally for authors and reviewers so that model evaluations can be as fair as possible.

Model publishing essentially comes down to conveying each of the previous nine steps to the audience in a structured fashion (Otto and Day, 2011; Kording and Mensh, 2016). The introduction section should describe the phenomenon/question that the model addresses (Step 1), provide relevant background information from the literature review (Step 2) and maybe introduce some of the ingredients needed (Step 3) as well general hypotheses (Step 4). Methods will detail all model ingredients (Step 3) and hypotheses (Step 4), justify the choice of the toolkit (Step 5) to answer the question asked and meet the goals. The final graphical draft of the model (Step 6) typically becomes the first detailed figure implementation (Step 7) as well as the procedures of model testing and evaluation (Step 9), which will also be detailed in the Methods section. Results will summarize model performance (Step 8) and provide the testing and evaluation statistics (Step 9) along with answering the original question (Step 1) and speaking to each of the specific and general hypotheses (Step 4). Thus overall, following the 10 steps of modeling also streamlines and simplifies the publishing step, especially if detailed notes have been taken all along the way.

Finally, there are a series of important guidelines to respect when publishing models:

- Know the target audience. Write in a way that your audience can understand. In most cases, the target audience should be experimentalists!
- In order for a model to receive the appropriate appreciation, it is absolutely crucial to clearly describe what the goals, hypotheses, and performance criteria were (Kording et al., 2018)!
- A model should always be graphically represented (Rougier et al., 2014) if at all possible.
- Show model behavior in parallel (i.e., side by side or superimposed) with the data that the model was designed to explain. This is a powerful way to prove to the research community that the model mechanisms have been correctly interfaced with the produced behavior.
- Publish the model code. Clean up the code and make it readable and understandable to others. Ideally, the published code should reproduce all figures of the results in the article. Publishing the code hugely increases the usefulness of the model for science (Prlić and Procter, 2012). Consider ModelDB (https://senselab.med.yale.edu/modeldb/) or similar repositories to publish your model.
- Publish the data that you fit your model to in one of the relevant databases (e.g. crcns.org, figshare, OSF.io).

## Discussion

We have argued that following these 10 simple steps should leave modelers with little room for failure. As mentioned before, we have successfully applied this pipeline to 2-week-long, small-group, model-building exercises at CoSMo. It is worth pointing out that this success occurred irrespective of model type or class (i.e., it worked for models ranging from neural networks to first principle derivations of normative behavior, and from model-driven data analysis to pure theory). Of course, for each type of question/model, the extent and practical implementation of the different how-to-model steps might looks different and be more or less extensive. However, importantly, all steps tend to apply to all types of modeling approaches.

### What is a good model?

Consider that you have done everything right, as outlined in the 10 easy steps to modeling. You framed the question precisely, had specific testable hypotheses, choose the right toolkit, implemented the model, fit it to data, selected the right number of parameters/the best model, cross-validated your results, and compared your best model to alternatives from the literature. Does that mean your model is a good model? In fact, what are the criteria of a good model?[Boxed-text box2]

“All models are wrong, but some are useful” (Box, 1976)

“The words true model represent an oxymoron” (Anderson and Burnham, 2002)

“Everything should be made as simple as possible, but no simpler” (Einstein)

There are many potential criteria motivating the development of a model, and many of them are valid criteria in judging whether the goals have been achieved (Kording et al., 2018). Criteria could be: explain data, interface with data, generalizes within sample/out of sample, robustness, reproducibility, bridging fields, across-fields predictions, interpretability, inspires experiments, clinical relevance, falsifiability, mechanistic insight, people care (funding), new predictions, technological applications, intervention/policy implications, nonarbitrary structure (elegance), subsumes previous models/data (unification), self-consistency, plausibility of hypotheses, simplicity, computing efficiency, realism, and normativity. Evidently, not all models satisfy all those criteria; in fact, satisfaction of any single criterion might be sufficient to consider the model as being of value. The precise choice of the evaluation criterion should be dictated by the modelers stated goals and the consensus of the field on how to evaluate such a goal is met. However, one universally important aspect about modeling is the subsumption principle (i.e., a good model should capture all existing phenomena in a domain, not just the data in front of the modeler).

Depending on the model criteria (see above), questions, and goals, a different model toolkit might be chosen to explain the same phenomenon. This is because different toolkits allow the answering of different types of questions and the achievement of different modeling goals (Kording et al., 2018; Blohm et al., 2019). As a result, models vary greatly along many dimensions, such as granularity (David Marr’s computational, algorithmic, and physical/implementation levels), generality (Peter Dayan’s and Larry Abbott’s descriptive, mechanistic, and interpretive models), or scale (physical extent of the system modeled). Depending on where is a model is situated in this high-dimensional model space, there are typically different constraints, scopes, and evaluation criteria for a model. It is thus useful to know where a model is situated in this space as it constrains the goals and defines the limitation of a model (Blohm et al., 2019).

### Good modeling practices

Meaningful model development in neuroscience should go hand in hand with good modeling practices. For example, iteratively modifying the model structure to obtain a better fit to the data are often done; however, this is not always advised because changing the model structure might imply changing the hypotheses on the fly, which is essentially HARKing (“Hypothesis After Results” justification). Furthermore, preregistration might prevent some of the biases in model comparison that stem from researchers’ motivation to show that their new model fits data better than previous models. Following our 10 simple rules in the correct order (Fig. 3) guards against this (often involuntary) fallacy. We strongly advise not making any changes to the model hypotheses and structure after Steps 1–6 have been completed. One good way to stay honest would be to preregister (Nosek et al., 2018) the model plan, outlining the hypotheses and test strategies developed in Steps 1–6. This does not prevent researchers from performing crucial adjustments to their models if initially hypothesized models fail to produce the expected result. Crucially though, preregistration “forces” authors to report the iterative adjustments, allowing the community to benefit from the insights gained throughout the process. For example, one could imagine a situation under which the hypothesized purpose of the clock would be to predict the movement of the stars; knowing this is wrong would help the community move forward in understanding the clock. Note that preregistering the modeling study in itself is to be considered separately from preregistering potential experimental predictions that result from the model. In summary, we suggest that preregistration of modeling efforts would lead to a cleaner, more comprehensive and reproducible model-building process in which logical steps and reasoning are clearly outlined and reproducible.

There might be limitations to when a modeling study should be preregistered. The above procedure might be most suitable when a model is a specific implementation of a hypothesized mechanism to explain previously described phenomena for which there are data. It might make less sense when the modeling effort consists in developing new theoretical tools or general theories (e.g., a new machine-learning approach or a new principled way of learning). However, we would argue that these exceptions are rather rare in neuroscience research compared with the abundance of models that directly target data.

## Conclusion

This 10-step pipeline has been proven to remove some of the apparent arbitrariness of the neuroscientific modeling process and to provide teachable instructions on how to succeed in modeling. Indeed, modeling currently looks much like a fashion show with the whims and trends dictating what is hot. This arbitrariness in the modeling approach may also lead to misguided model judgments. We emphasize that modeling is not a beauty contest; models need to be judged based on their well defined goals, not their appearance or fashionability. To allow fair judgment, authors have the responsibility to clearly lay out their thought process. While this 10-step guide is tailored toward the neuroscience community, it should help achieve this goal throughout life sciences and beyond.[Boxed-text box3]

Example box

Modeling eye movements

David A. Robinson is generally considered the father of quantitative oculomotor research. Here, we will use one of his most influential modeling studies (Robinson, 1973: Models of the saccadic eye movement control system) as an example illustrating our 10 steps “how to model.”

**Figure 5.**
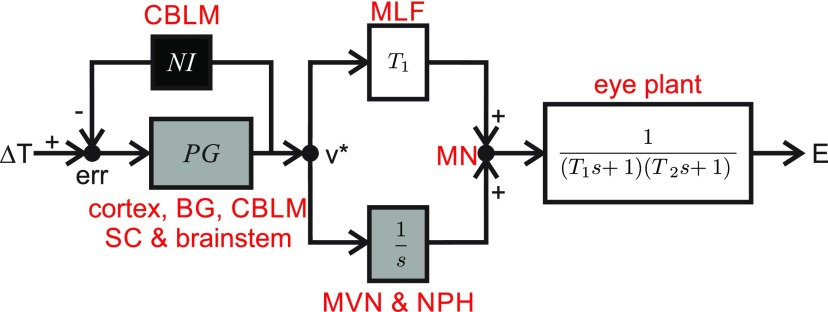
Updated version of Robinson’s simple saccade model (Scudder, 1988). Saccade target shift (ΔT) is compared to an internal estimate of saccade progression computed through the resettable neural integrator [NI, suggested by Scudder (1988), not Robinson] to provide a motor error (err). Based on circumstantial evidence, Robinson’s insight led him to postulate the pulse generator (PG) to provide a desired eye velocity drive (v*). This pulse command was scaled to match the eye plant dynamics (gain, $T_{1}$) and provided the saccade drive. However, Robinson recognized that viscoelastic forces would pull the eye back to primary position if not actively compensated for. This is how he proposed the neural integrator ($\frac{1}{s}$) to provide a tonic drive that overcomes the viscoelastic forces. Tonic and phasic drives add up and are sent to extraocular muscles of the eye plant that he modeled as a second-order system to move the eye (E). Red labels are mappings of individual computations to specific brain areas. CBLM, Cerebellum; BG, basal ganglia; SC, superior colliculus; MLF, medial longitudinal fasciculus; MVN, medial vestibular nucleus; NPH, nucleus prepositus hypoglossi; MN, motor neurons. Gray boxes indicate Robinson’s innovations. Black box denotes a later modification of Robinson’s model by Scudder (1988), included here for correctness.

- ***Step 1***: In general, Robinson asked whether we can understand the neural organization that controls saccadic eye movements by establishing relationships between computations in an abstract controller and the activity in subcortical brain areas, such as motor nuclei. In doing so, he is really addressing two different questions. (1) Are eye movements expressible as the result of an abstract controller (causal question)? And (2) is the neural activity compatible with latent variables in an abstract controller (explanatory question)? For the latter, Robinson referred to novel specific data from oculomotor nuclei.

- ***Step 2***: Robinson grounds his model in the literature, using a previously published and highly influential model of the extraocular muscle and eye ball mechanics (Robinson, 1964) as a starting point for his oculomotor controller. He could also rely on electrophysiological recordings in oculomotor neurons (Robinson, 1964, 1970) as well indirect evidence for a neural integrator in the eye premotor circuitry (Skavenski and Robinson, 1973). Finally, he obtained crucial intuitive insight from the stereotypical nature of saccadic eye movements, specifically the high degree of regularity of their velocity profile.

- ***Step 3***: Since Robinson was interested in producing eye movements to a target, model input is an abstract motor goal and model output is eye position (Fig. 5). How did Robinson choose the right variables? How did he make sure that these variables were compatible with the phenomenology in terms of magnitude, resolution (level of detail), and timescale? It was known from oculomotor neuron electrophysiology that the eye plant needed a pulse and a step command to overcome elastic and viscous forces, respectively. Robinson’s model needed to generate such neural commands as latent variables and used a neural integrator to produce the step from a pulse. Finally, he needed a pulse generator that was able to convert a motor error (or goal) into a pulse that could then drive the saccade. He was only interested in reproducing average population firing rates, not single action potentials. He also only considered eye movements starting from the primary eye position (Fig. 5).

- ***Step 4***: Robinson hypothesized that saccades result from a pulse input to the ocular plant. He also hypothesized that a neural integrator existed and that it integrated a scaled version of the pulse command. Pulse and step commands should then be added up again at the level of the motor neurons (Fig. 5).

- ***Step 5***: Robinson used linear control systems theory as a toolkit to address his question because he believed that the brain needed to implement some natural neural control law and he knew that any such dynamical system could be locally well approximated by a linear system (see goals, Step 1). In choosing this toolkit, he hoped to span all three levels of Marr from computational (i.e., overall system behavior describing eye movements) to algorithmic (i.e., how this behavior could be implemented most efficiently) to physical (i.e., neural population coding of the individual components of his model).

- ***Step 6***: Robinson drew a draft diagram of the model given his knowledge and hypotheses (Fig. 5). He could then fill in the boxes using linear control theory language. For example, his hypotheses allowed him to write down a potential premotor circuit transfer function. He also already knew the transfer function of the eye plant from his previous work. Finally, he needed a pulse generator. Since little was known about it, he chose what he thought was the simplest arrangement reproducing the correct saccade dynamics. Note that Robinson also chose all his latent variables in his model to represent observable firing rates of real neural areas.

- ***Step 7***: Robinson’s first model was elegant in that it used known physiology to produce saccadic eye movements in a seemingly simple fashion. However, he knew that this model was unlikely to be able to reproduce other aspects of saccades or their neural control, such as saccades to moving targets. He (and other authors) therefore incrementally expanded his model in follow-up studies to include missing aspects.

- ***Step 8***: Robinson considered his task achieved when his models were able to qualitatively reproduce the specific data he set out to model. He thereby answered his two initial questions: that latent variables in his model are indeed consistent with oculomotor electrophysiology; and that linear control systems theory could accurately capture the control of eye movements of the brain, at least in the brainstem.

- ***Step 9***: Robinson only carried out qualitative model evaluations. This included comparing model and real eye movement behavior as well as comparing model predictions of latent variables to neuronal recordings. Nowadays, reviewers would probably encourage him to provide more quantitative comparisons with eye movement data as well as a critical evaluation of his models with other existing ones, but scientific standards were different in 1973. However, his model made very interesting predictions regarding the presence of a common neural integrator for all eye movements as well as a phasic (pulse) motor command. Since Robinson’s eye plant model in 1964, he also believed that principles of linear control theory can be used to describe all eye movements, which led to half a century of extremely fruitful theoretical and experimental work (breadth of application). As a result of his model-driven approach, the eye movement system is now arguably the best understood neural system.

- ***Step 10***: Robinson published his manuscript in a journal called *Kybernetik* (nowadays *Biological Cybernetics*), which is mostly targeted toward engineers trying to understand biological systems. He clearly laid out his goals, described all details of his approach, and related his findings to experimental data. But, enough said; we encourage the reader to generate his/her own opinion by reading Robinson’s article.

## Synthesis

Reviewing Editor: Li Li, New York University Shanghai Decisions are customarily a result of the Reviewing Editor and the peer reviewers coming together and discussing their recommendations until a consensus is reached. When revisions are invited, a fact-based synthesis statement explaining their decision and outlining what is needed to prepare a revision will be listed below. The following reviewer(s) agreed to reveal their identity: Zhong-Lin Lu.

Both reviewers agreed that the ms can be considered for publication once the authors take care of their concerns. I list their comments below:

Reviewer 1

This is a well written piece on how to model in neuroscience. I have two comments for the authors to consider: (1) Although the paper may have covered systematic ways to model data in neuroscience in most situations, there are other ways to create models, e.g., those based on first principles. (2) Another point that might be worth more emphasize is the scope of a model. A good model must be able to explain all the existing phenomena in its domain, not just the data in front of the modeler.

Print Report.

Reviewer 2

This paper outlines a set of recommendations for model building in neuroscience. Given the importance of models in our understanding of neural computation, a clear, concise, and sensible guide would be beneficial to the field. I think the authors have done a nice job outlining such a framework. It might be case that the individual principles outlined in the paper are already known, but putting them together in a coherent framework would still be useful as a reference.

I only have some minor suggestions for the authors to consider.

1. Models vary greatly on their details and one useful dimension to consider is Marr’s distinction of levels. It seems to me that algorithmic models (e.g., linear system, sequential accumulation) and implementational models (e.g., neural network, receptive field) could have some different considerations. The latter needs to consider neurophysiological findings more seriously in model construction. For example, if a model consists of sensory neurons tuned to a feature (e.g., orientation), then the shape of the tuning curves are somewhat constrained by physiology, whereas more abstract models have less such constraints. I wonder if this is a point worth discussing.

2. It appears that the authors were talking about constructing one model. Is it recommended that we should construct multiple models? In the model testing section (line 466), model comparison was mentioned. But that requires at least two models to compare with. I wonder what the authors’ take on this is.

3. In the second paragraph of the Discussion the authors listed many potential criteria to evaluate models. Is there any consensus that some of these are more important than others? If so it would be very useful for practitioners to know. At least I hope there are some ways to rank order them; or perhaps different criteria have different weights depending on the type of the model? Some more guidance would be helpful.

4. The concept of ‘lumpable’ is not really explained (line 351). I think for the general audience of people interested in modeling, this should be explained in plain language.

5. There are a number of typos:

line 348: ‘we could we could’

p. 19, 2nd paragraph, “indiace”

p. 19, 4th paragraph, “natural natural”
